# Supplementary figures and images for: A high-quality de novo genome assembly based on nanopore sequencing of a wild-caught coconut rhinoceros beetle (Oryctes rhinoceros)
Source: BMC Genomics. 2022 Jun 7;23:426. doi: 10.1186/s12864-022-08628-z (PMC9172067; doi:10.1186/s12864-022-08628-z)

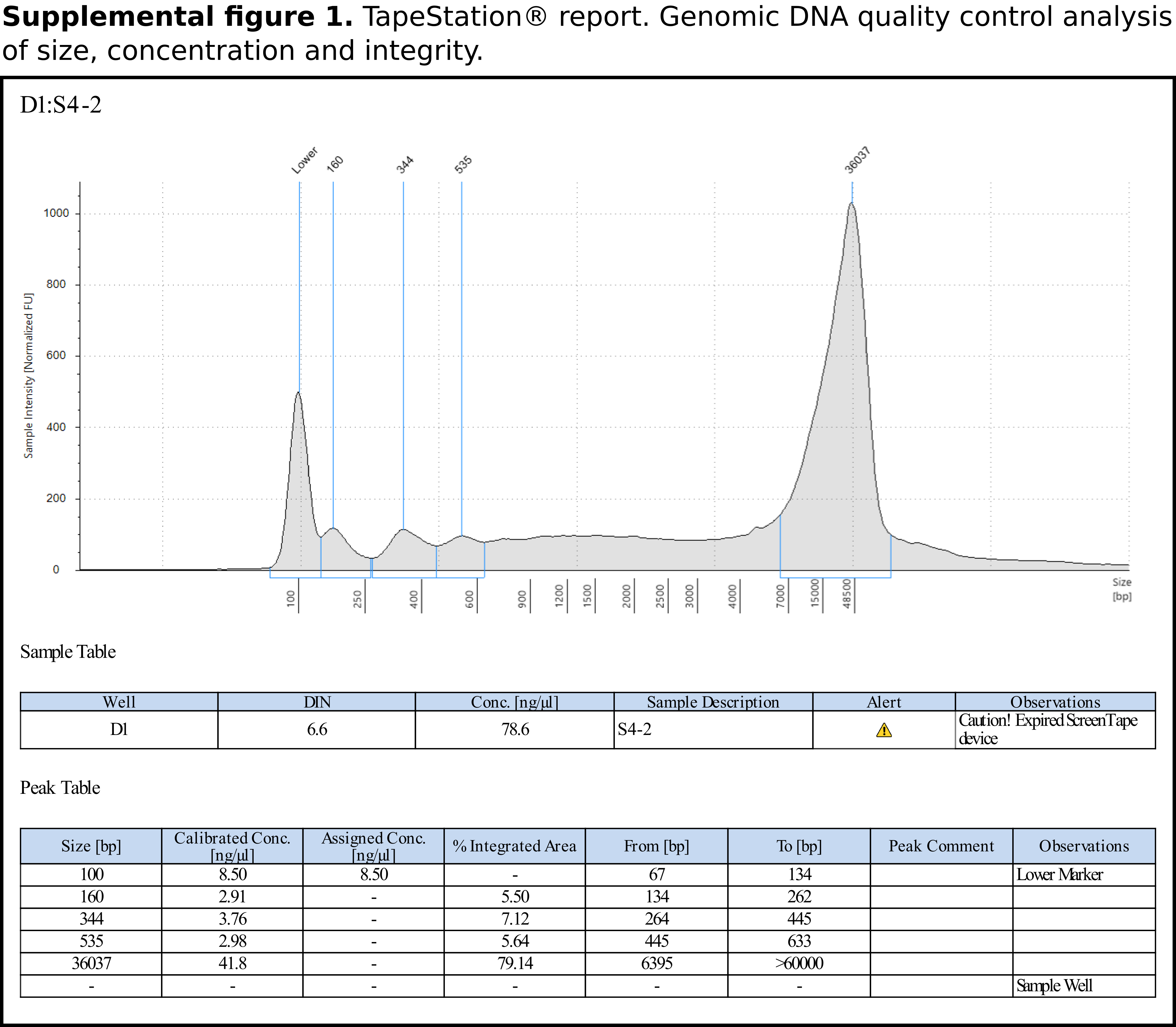

Supplement: Supplementary file 6 — Additional file 6: Supplemental Figure 1. TapeStation® report. Genomic DNA quality control analysis of size, concentration and integrity. [file 12864_2022_8628_MOESM6_ESM.png]
